# Three new species of the genus *Philopteroides* Mey, 2004 (Phthiraptera, Ischnocera, Philopteridae) from New Zealand

**DOI:** 10.3897/zookeys.297.5118

**Published:** 2013-05-10

**Authors:** Michel P. Valim, Ricardo L. Palma

**Affiliations:** 1 Museu de Zoologia da USP, Av. Nazaré, 481, Ipiranga, São Paulo, SP 04263-000, Brazil; 2 Museum of New Zealand Te Papa Tongarewa, P.O. Box 467, Wellington, New Zealand

**Keywords:** *Philopteroides*, Philopteridae, Phthiraptera, lice, new species, new combination, key to species, Passeriformes, New Zealand

## Abstract

We describe and illustrate three new species of chewing lice in the genus *Philopteroides* parasitic on passerines (Order Passeriformes, families Acanthizidae, Rhipiduridae and Petroicidae) from New Zealand. They are: *Philopteroides pilgrimi* **sp. n.** from *Gerygone igata igata*; *Philopteroides fuliginosus* **sp. n.** from *Rhipidura fuliginosa placabilis* and *Rhipidura fuliginosa fuliginosa*; and *Philopteroides macrocephalus* **sp. n.** from *Petroica macrocephala macrocephala* and *Petroica macrocephala dannefaerdi*. The identity of *Docophorus lineatus* Giebel, 1874 is discussed based on its morphology and host association. We also transfer *Tyranniphilopterus beckeri* to the genus *Philopteroides*, and provide a key to identify adults of 12 of the 13 species now included in *Philopteroides*.

## Introduction

The genus *Philopteroides* was erected by Mey (2004) to include seven species, three of which he described as new and four of which he transferred from other genera. Najer and Sychra (2012a, b) described two additional species, and we herewith describe three further new species from New Zealand, as well as transfer a species described in the genus *Tyranniphilopterus* by Mey (2004) to *Philopteroides*, bringing the total number of species to 13. We also give additional diagnostic characters to distinguish *Philopteroides* from closely related genera included in the *Philopterus*-complex (*sensu* Mey 2004).

Species of the *Philopterus*-complex are relatively sedentary lice belonging to the *docophorid ecotype* (Mey 2004) and highly adapted to live on feathers of the host’s head & neck, on which they spend their entire life cycle. The hosts of the 13 species of *Philopteroides* belong to several families within the avian order Passeriformes, covering a wide geographical distribution over Africa, Asia and Oceania (see below).

## Methods

Specimens examined from New Zealand hosts belong to the Museum of New Zealand Te Papa Tongarewa, Wellington, N.Z. (MONZ), except for some paratypes deposited in the Museu de Zoologia da Universidade de São Paulo, São Paulo, Brazil (MZUSP). The remaining material examined is held in the Naturhistorischen Museum, Rudolstadt, Thüringen, Germany (NHMR). The lice were treated and mounted on slides following the Canada balsam technique (Palma 1978).

All measurements are in millimeters, taken from digitalized images from slide-mounted specimens using the software *Leica* Application Suite (LAS), and identified by the following abbreviations: *as3*, length of the anterior setae 3; ADPL, anterior dorsal plate length (taken at middle line); ADPLL, anterior dorsal plate lateral length (taken from the base of anterior dorsal setae – *ads*, to lateral apices of the plate); ADPW, anterior dorsal plate width (taken at its widest point); ANW, width of anterior notch (taken between the bases of *as3*); AW, maximum width of the abdomen (taken at level of segment V); EWG, external width of genital chamber; GL, male genitalia length; GW, male genitalia width (taken at the basal plate); HL, head length (excluding hyaline membrane); IWG, internal width of genital chamber; POL, preantennal length (taken from the base of the conus to the bases of *as3*, obliquely to the head axis); POW, preantennal width (taken between bases of the coni); PTW, pterothorax width; PTL, pterothorax length; PW, prothorax width; TL, total length; TPVL, tergal plate V length; TRL, trabecula length; TRW, trabecula width; TW, temporal width.

The sternal abdominal setae are given in sequence from the left to the right side and named by the letters: “L” meaning long and flexible, and “S” meaning short and spine-like. Thus, the first letter indicates the outermost seta on the left side and the last letter the outermost on the right side. For example: LSLS-SLSL if the pattern is symmetrical, or LSLS-L-SLSL if asymmetrical.

The minute spine-like and the long trichoid setae present on each side of the pterothorax are not included in the number of pterothoracic setae. The chaetotaxy of the abdominal tergocentral setae does not include the postspiracular setae, except for tergite II where postspiracular setae, if present, can not be distinguished from the remaining setae. We regard as pleural setae those situated on the lateral sides of the tergo-pleurites, and as sternal setae those next to the sternites. Some species have ventral setae between the innermost pleural and the outermost sternal setae, which we regard as additional setae.

The nomenclature of head features and setae follows Clay (1951), as amended by Mey (1994). Scientific nomenclature, vernacular names, and the classification of hosts follow those in Dickinson (2003).

## Taxonomic treatment

### Philopteroides

Mey, 2004

http://species-id.net/wiki/Philopteroides

#### Type species.

*Philopteroides novaezelandiae* Mey, 2004 (by original designation).

**Two species-groups:**

*beckeri* species-group: two species

*mitsusui* species-group: ten species

#### Host distribution.

Passeriformes, suborder Acanthisitti (Acanthisittidae), and suborder Passeri (Acanthizidae, Meliphagidae, Monarchidae Nectariniidae, Petroicidae, Platysteiridae, Pycnonotidae, Rhipiduridae).

#### Geographical range.

Africa (Senegal, Uganda), Asia (India, Vietnam, Taiwan), Oceania (Micronesia, New Zealand).

In addition to those characters mentioned by Mey (2004) in his original description of the genus, we add further diagnostic characters to support the generic position of *Philopteroides* within the *Philopterus*-complex.

#### Diagnosis.

Member of the *Philopterus*-complex by presence of well-developed trabeculae. Anterior dorsal head plate with posterior median projection well developed and sclerotized, but without antero-lateral projections. Hyaline membrane and anterior head plates deeply concave forming an “osculum” (*mitsusui* group) (Figs 11–12, 34, 36); some species with wide frons (*beckeri* group) (Figs 9–10). Hyaline membrane deeply or slightly concave, arising from the level of the tips of the marginal carinae or above the anterior setae 3 (*as3*), with a conspicuous median sclerotization and without additional setae. Marginal carina not interrupted laterally, but with a conspicuous lateral suture on the dorsal surface, at the level of the posterior dorsal sub-medial setae (*d.sm.s*.). Conus ranging from much reduced to well developed. Marginal temporal setae 2 (*m.t.s.2*) and pre-ocular setae (*p.o.s*.) median to short. Prothoracic dorsal setae close to the middle of the segment, and to its posterior margin. Pleuro-tergal plates II–IV without postero-lateral projections (‘posterior heads’), but few species with at most a slightly pronounced angle on segment II, but not on III or IV. Spine-like setae present on some of the sternites II–VI.

**Figures 1–4.**
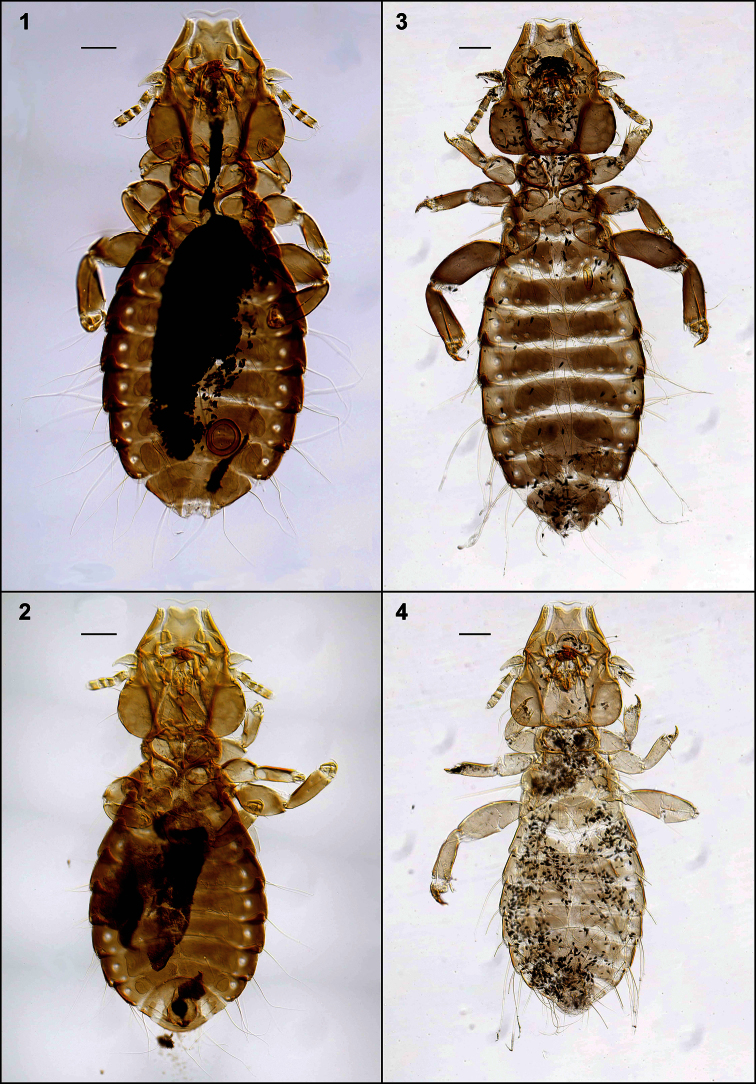
Habitus, ventral views: *Philopteroides beckeri* female paratype (**1**); *Philopteroides beckeri* male holotype (**2**); *Philopteroides pilgrimi* female holotype (**3**); *Philopteroides pilgrimi* male paratype (**4**). Scale bars = 0.1 mm

**Figures 5–8.**
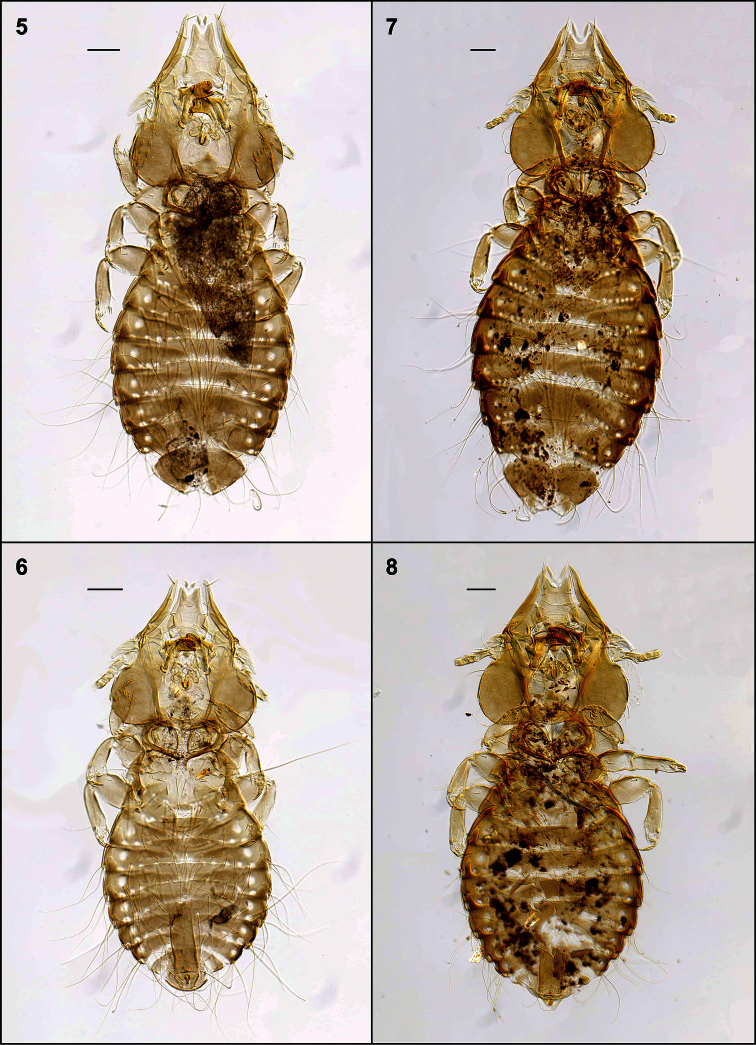
Habitus, ventral views: *Philopteroides fuliginosus* female holotype (**5**); *Philopteroides fuliginosus* male paratype (**6**); *Philopteroides macrocephalus* female holotype (**7**); *Philopteroides macrocephalus* male paratype (**8**). Scale bars = 0.1 mm

**Figures 9–12.**
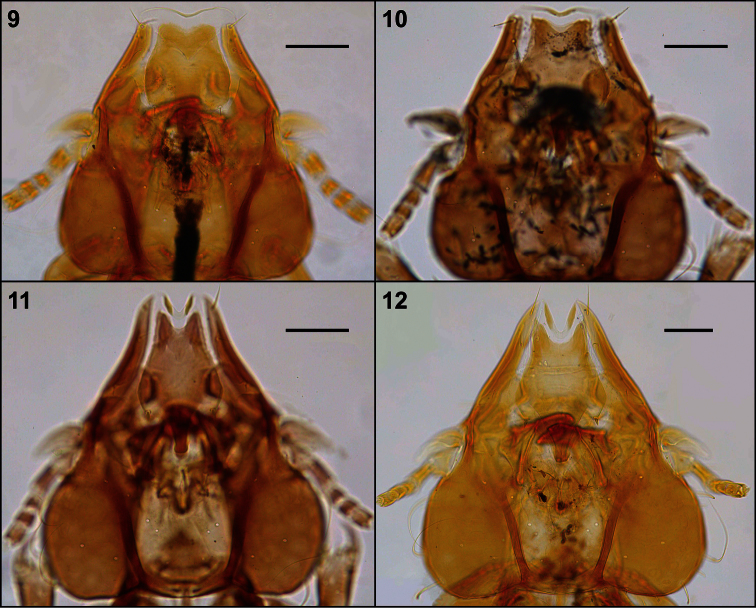
Head, dorsal view: *Philopteroides beckeri* female paratype (**9**); *Philopteroides pilgrimi* female holotype (**10**); *Philopteroides fuliginosus* female (**11**); *Philopteroides macrocephalus* female holotype (**12**). Scale bars = 0.1 mm

**Figures 13–20.**
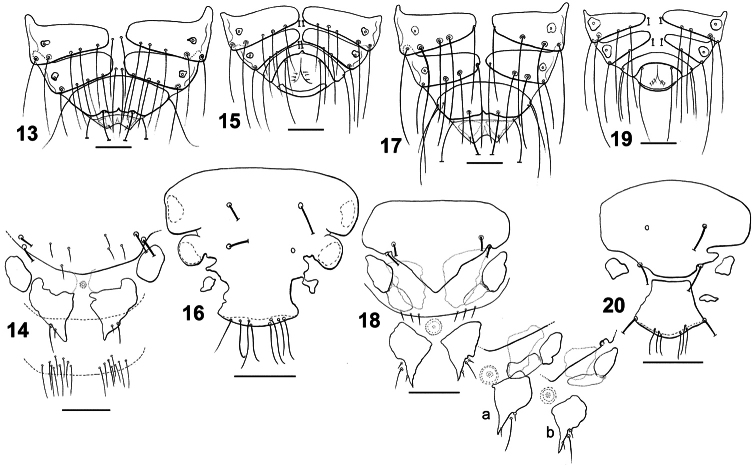
Dorsal terminalia: *Philopteroides beckeri* female (**13**); *Philopteroides beckeri* male (**15**); *Philopteroides pilgrimi* female (**17**); *Philopteroides pilgrimi* male (**19**). Female ventral terminalia: *Philopteroides beckeri* (**14**); *Philopteroides pilgrimi* (**18**); *Philopteroides pilgrimi* intraspecific variation (**18a,b**). Male subgenital plate: *Philopteroides beckeri* (**16**); *Philopteroides pilgrimi* (**20**). Scale bars = 0.1 mm.

**Figures 21–28.**
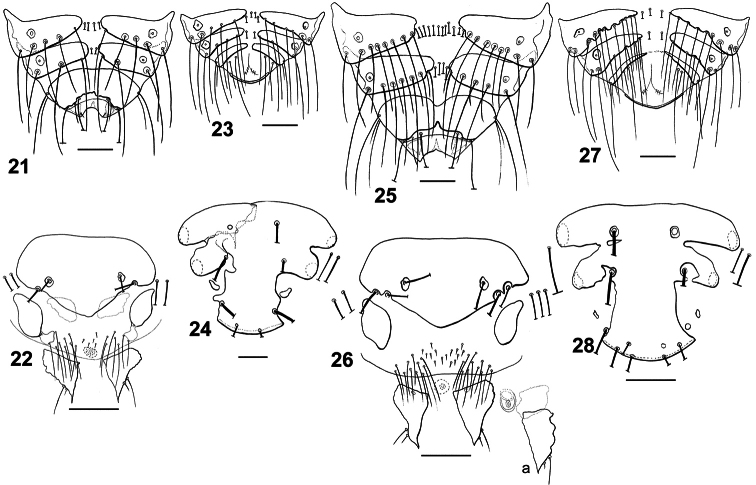
Dorsal terminalia: *Philopteroides fuliginosus* female (**21**); *Philopteroides fuliginosus* male (**23**); *Philopteroides macrocephalus* female (**25**); *Philopteroides macrocephalus* male (**27**). Female ventral terminalia: *Philopteroides fuliginosus* (**22**); *Philopteroides macrocephalus* (**26**); *Philopteroides macrocephalus* intraspecific variation (**26a**). Male subgenital plate: *Philopteroides fuliginosus* (**24**); *Philopteroides macrocephalus* (**28**). Scale bars = 0.1 mm.

**Figures 29–37.**
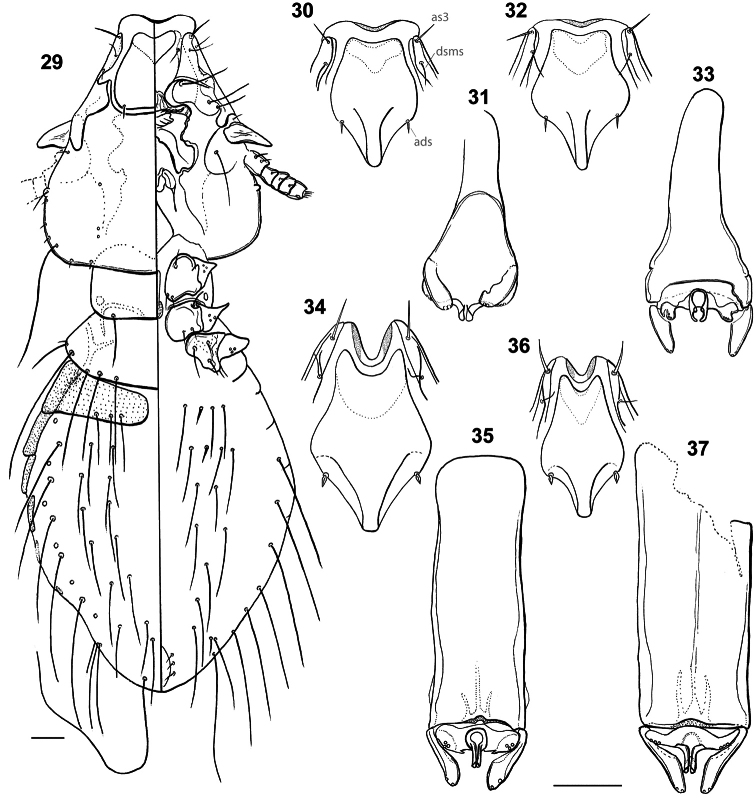
Habitus: *Philopteroides beckeri* nymph II, dorso-ventral view (**29**). Hyaline margins and anterior dorsal plates: *Philopteroides beckeri* (**30**); *Philopteroides pilgrimi* (**32**); *Philopteroides fuliginosus* (**34**); *Philopteroides macrocephalus* (**36**). Male genitalia: *Philopteroides beckeri* (**31**); *Philopteroides pilgrimi* (**33**); *Philopteroides fuliginosus* (**35**); *Philopteroides macrocephalus* (**37**). Scale bars = 0.05 mm

#### Note.

*Docophorus lineatus* Giebel, 1874 was included in *Philopteroides* by Mey (2004: 174), when he described it as a new genus. Considering that Giebel’s types of this species were lost during the Second World War, and that we have not been able to examine any material from the type host, we follow Mey (2004) in including *Docophorus lineatus* in *Philopteroides*, but we are unable to place it in any of the two species-groups which we define below. A neotype for *Docophorus lineatus* is urgently needed to clearly define this species.

### Philopteroides lineatus

(Giebel, 1874)

http://species-id.net/wiki/Philopteroides_lineatus

Docophorus lineatus Giebel, 1874: 91.
Philopterus lineatus (Giebel, 1874); Harrison 1916: 98; Hopkins and Clay 1952: 285; Price et al. 2003: 215.
Philopteroides lineatus (Giebel, 1874); Mey 2004: 174.

#### Type host.

*Arachnothera longirostra* (Latham, 1790) – Little spiderhunter (Nectariniidae). See note below.

#### Distribution.

Unknown. The original description does not include a type locality. There are 13 subspecies of *Arachnothera longirostra* distributed throughout the Indo-Malayan region (Dickinson, 2003).

#### Remarks.

Considering current louse species descriptions, Giebel’s (1874) description of *Docophorus lineatus* is superficial and without any illustration. However, from Giebel’s original comparison of this species with *Docophorus communis* (= *Philopterus citrinellae* (Schrank, 1776) ), and in agreement with Harrison (1916), Hopkins and Clay (1952) and Price et al. (2003), we believe, without doubt, that *Docophorus lineatus* belongs to the *Philopterus*-complex.

In his original description, Giebel (1874) clearly states “... Schläfenecke drei sehr lange über den Prothorax hinausragende” (= “... *marginal temporal carina with three very long macrochaetae projecting beyond the prothorax*”). This feature is present in species of *Philopterus* Nitzsch, 1818 sensu stricto, *Clayiella* Eichler, 1940, and in a few species of *Mayriphilopterus* Mey, 2004, but not in species of *Philopteroides* which have, at most, two long marginal temporal setae (*mts*), with the *mts2* always short (Mey 2004). Hence, it would appear that *Docophorus lineatus* is not a *Philopteroides* sensu stricto, unless Giebel (1874) included the ocular seta – which is medium to long in *Philopteroides*– in his three long marginal temporal setae, but the ocular seta does not project beyond the pronotum. Therefore, until material from the type host becomes available to allow for a neotype designation, it is not possible to establish with certainty the correct generic position of *Docophorus lineatus* within the *Philopterus*-complex.

Regarding the host-louse association of *Docophorus lineatus*, Giebel (1874) gave the type host as “*Arachnothera (Certhia) longirostris*”. Hopkins and Clay (1952: 285) interpreted that host species as being conspecific with “*Certhia b. brachydactyla* Brehm”, according to the bird nomenclature of the time. However, Dickinson (2003: 714) listed *Arachnothera longirostra* (Latham, 1790) as a valid species in the family Nectariniidae, as well as *Certhia brachydactyla* Brehm, 1820 as a valid species in the family Certhiidae (Dickinson 2003: 648). In our opinion, the name of the species of the type host given by Giebel (1874) is the most important piece of information to establish the correct type host, regardless of the genus or subgenus associated with that species. Therefore, in agreement with Mey (2004: 174, footnote), we consider that the type host of *Docophorus lineatus* is *Arachnothera longirostra* (Latham, 1790). The fact that another species of *Philopteroides* has a type host in the family Nectariniidae (see *Philopterus sclerotifrons* Tandan, 1955 below) is further evidence that (1) *Arachnothera longirostra* is the correct type host for *Docophorus lineatus* and (2) *Philopteroides* may be the correct genus for *Docophorus lineatus*. Furthermore, there is no species of *Philopteroides* recorded from any member of the Certhiidae.

##### *beckeri* species-group

The trapezoidal shape of the head is a distinctive character in the two species of this group. The preantennal region is short (POL 0.15–0.18) and broad (ANW 0.13–0.15), with a hyaline margin shallowly concave at midline. Conus very reduced.

### Philopteroides beckeri

(Mey, 2004) comb. n.

http://species-id.net/wiki/Philopteroides_beckeri

Figs 1–2 , 9 , 13–16 , 29–31

Tyranniphilopterus beckeri Mey 2004: 182; Cicchino 2007: 49.

#### Type host.

*Platysteira cyanea nyansae* Neumann, 1905 – Brown-throated wattle-eye. (Platysteiridae)

#### Distribution.

Uganda.

This species was recently described in detail and, therefore, it is not necessary to redescribed it again. We only include habitus images of the holotype male and one paratype female (Figs 1–2), not figured in the original description by Mey (2004). We also include illustrations to support our change of the original generic combination of this species, and have re-drawn only those characters (Figs 9, 13–16, 30) useful to distinguish it from the second species described below. In addition, the second nymphal stage is described from a single specimen (Fig. 29) mounted together with the female paratype, and a re-interpretation of the male genitalia is presented in Fig. 31.

All species of the genus *Tyranniphilopterus* Mey, 2004 have the following characters, which are lacking in “*Tyranniphilopterus*” *beckeri*: (1) hyaline margin arises at a level below the *as3*; (2) conspicuous antero-lateral projections on anterior dorsal head plate; (3) prothoracic dorsal setae located on the posterior-lateral angles of the segment, not on its posterior margin.; (4) pleuro-tergal plates II–III with well developed postero-lateral projections. Instead, “*Tyranniphilopterus*” *beckeri* has the features which define *Philopteroides*. These morphological features justify placing it in the latter genus, as *Philopteroides beckeri* (Mey, 2004) new combination.

Furthermore, the hosts of all species of *Tyranniphilopterus* – except for “*Tyranniphilopterus*” *beckeri* – belong to the passerine suborders Tyranni and Passeri, and are confined to the Americas, while the hosts of all species of *Philopteroides* belong to the passerine suborders Passeri and Acanthisitti distributed in Africa, Asia and Oceania. Hence, the geographical distribution of its host is further evidence that placing “*Tyranniphilopterus*” *beckeri* in the genus *Philopteroides* is correct.

#### Description of second nymphal stage.

Head, thorax and abdomen as in Fig. 29. Head sub-conical, marginal carina entire laterally, with well developed anterior dorsal and ventral head plates. Anterior setae 3 (*as3*) rigid, 0.04 in length. Dorsal head plate with convex lateral margins and almost straight posterior margin, bearing rigid anterior dorsal head setae (*ads*), as the adults. Anterior ventral plate cordiform. Marginal temporal setae 3 (*m.t.s.3*) very long, other temporal setae short to minute. Each of the pair of posterior setae on pronotum located half way between the middle of the segment and its lateroposterior angle. Only four long pterothoracic setae, as in the second nymphal stage of most species of Philopteridae (Mey 1994). Abdomen mostly membranous, except for the tergo-pleural plate of second segment (first visible), and the pleural plates of segments III–V. Segments VI and VII with small patches of light sclerotization. Abdominal chaetotaxy as in Fig. 29, with two long dorsal setae plus the postspiracular seta on each side of segments III–VIII. Sternites II–III with one spine-like setae among three long setae on each side, IV–V with a pair of long setae each side, and VI–VIII with only one long setae on each side. One long pleural seta on III–VIII. Measurements (n=1): HL 0.37, ANW 0.11, POL 0.14, POW 0.27, ADPL 0.12, ADPW 0.14, TRL 0.08, TRW 0.04, TW 0.36, PW 0.21, PTW 0.29, AW 0.43, TL 1.06.

#### Type specimens.

Holotype ♂ (NHMR #4322.c) and 1♀ paratype (NHMR #4322.b), ex *Platysteira cyanea nyansae*; UGANDA: Paraa, Murchison Falls, National park, 22.X.1998, P. Becker col.

#### Non-type specimen.

1 nymph II (NHMR #4322.b), mounted on the same slide as the female paratype.

### Philopteroides pilgrimi

Valim & Palma sp. n.

urn:lsid:zoobank.org:act:22B8C261-0710-4C23-85B6-54EA58333235

http://species-id.net/wiki/Philopteroides_pilgrimi

Figs 3–4 , 10 , 17–20 , 32–33

#### Type host.

*Gerygone igata igata* (Quoy & Gaimard, 1830) – Grey warbler (Acanthizidae)

#### Distribution.

New Zealand.

#### Description.

**Female.** Habitus as in Fig. 3 and head as in Fig. 10. Anterior setae 3 (*as3*) rigid, 0.04–0.05 long, anterior dorsal head setae (*ads*) minute, 0.02 long. Hyaline membrane with shallow concavity and thin sclerotization; anterior dorsal plate slightly concave and ventral head plate deeply concave (Fig. 32). Pterothorax with 6–8 posteromarginal setae on each side. Tergo-pleural plate II with a reduced postero-lateral projection overlapping segment III, tergo-pleural plate III without projection. Tergocentral setae: II, 5–6 (plus 2 anterior setae); III, 6; IV, 5–6; V, 5–6; VI, 4–6; VII, 5–6; VIII, 5. Tergites VII–IX+X as in Fig. 17. Abdominal sternal setae: segment II, LS-SL; III, LS-SL; IV, SS-SS (rarely LS on one side only); V, SS-SS; VI, LL-LL (one female with one S on one side only). Paratergal setae (all long): II–III, 0; IV–V, 2; VI–VIII, 3. Tergites VI–VIII with an innermost seta (included in the paratergal count), lateral to postspiracular seta. Sternites III–VI well-developed as large, rectangular plates. Vulva with 3 medium long setae each side. Subgenital plate, inner genital sclerite, subvulval sclerites, and vulvar margin as in Fig. 18, and intraspecific variation of two additional females as in Figs 18a and 18b. Measurements (n = 8): HL 0.39–0.43, ANW 0.13–0.15, POL 0.16–0.17, POW 0.28–0.31, ADPL 0.21–0.22, ADPLL 0.14, ADPW 0.13–0.16, TRL 0.09–0.10, TRW 0.03–0.04, TW 0.35–0.41, PW 0.22–0.28, PTL 0.13–0.14, PTW 0.29–0.35, TPVL 0.19–0.25, AW 0.44–0.54, EWG 0.06–0.08; IWG 0.03–0.04, TL 1.35–1.65.

**Male.** Similar to female, except in dimensions and morphology of terminalia (Figs 4, 19, 20). Pterothorax with 5–6 posteromarginal setae on each side. Tergocentral setae: II, 6 (plus 2 anterior setae); III, 6; IV, 6; V, 6; VI, 6; VII, 6; VIII, 4. Dorsal terminalia as in Fig. 19. Sternal setae as for female. Paratergal setae (all long): II–III, 0; IV–V, 2; VI–VIII, 3. Sternal plates well developed and entire on segments III–VI; subgenital plate with 4 long setae as in Fig. 20, the anterior pair on the plate, and the posterior on the plate margin. Genitalia: length of parameres 0.40 (Fig. 33). Measurements (n = 1): HL 0.38, ANW 0.14, POL 0.15, POW 0.28, ADPL 0.18, ADPLL 0.13, ADPW 0.14, TRL 0.09, TRW 0.03, TW 0.36, PW 0.21, PTL 0.15, PTW 0.31, TPVL 0.22, AW 0.47, GL 0.19, GW 0.08, TL 1.30.

#### Type specimens.

Holotype ♀ (MONZ AI. 030137), ex *Gerygone igata igata*; NEW ZEALAND, no other data. Paratypes: 1♂, 2♀ (MONZ AI.017303) 1♀ (MZUSP #2885), same data as holotype; 2♀ (MONZ AI.017299), same host species, NEW ZEALAND: Orongorongo Valley, 20.V.1976, B.M. Fitzgerald col.; 1♀ (MONZ AI.017301), same host species, NEW ZEALAND: Kowhai Bush, Kaikoura, 15.XI.1976, B. Gill col.; 1♀ (MONZ AI.017302), same host species, NEW ZEALAND: Orongorongo Valley, 12.V.1977, B.M. Fitzgerald col..

#### Etymology.

This species is named in memory of the late Professor Robert L.C. Pilgrim (1921–2010), for his outstanding contribution to knowledge of ectoparasitic insects, and for his long friendship with RLP (Palma 2011).

#### Remarks.

Morphologically close to *Philopteroides beckeri*, especially by features of the head. In addition to the key characters mentioned below, the habitus of both species is distinct (compare Figs 1 and 3; 2 and 4, respectively). Both sexes of *Philopteroides pilgrimi* can be distinguished by (1) the presence of spine-like setae on sternite V (absent in *Philopteroides beckeri*); (2) female tergites IX+X with long innermost setae situated on the tergal plate (Fig. 17) (on soft tegument in *Philopteroides beckeri*, Fig. 13); (3) female subgenital plate without medial setae (Fig. 18) (against three pairs of setae on each side, as in *Philopteroides beckeri*, Fig. 14); (4) shape of sub-vulval sclerites (compare Figs 14 and 18); (5) male subgenital plates (compare Figs 16 and 20); and (6) male genitalia (compare Figs 31 and 33).

##### *mitsusui* species-group

The approximately triangular shape of the head is the distinctive character in the ten species included in this group. The preantennal region is longer (POL 0.22–0.29) and narrower (ANW 0.10–0.12) than in the *beckeri* species-group, and the hyaline margin is deeply concave at midline. Conus well-developed.

***Philopteroides mitsusui* (Uchida, 1948)**

*Bitrabeculus mitsusui* Uchida, 1948: 321, fig. 7.

*Philopterus mitsusui* (Uchida, 1948); Hopkins and Clay 1952: 286; Price et al. 2003: 215.

*Philopteroides mitsusui* (Uchida, 1948); Mey 2004: 174.

**Type host.***Myzomela rubratra dichromata* Wetmore, 1919 – Micronesian honeyeater (Meliphagidae)

**Distribution.** Pohnpei I., Caroline Islands, Micronesia.

***Philopteroides kayanobori* (Uchida, 1948)**

*Bitrabeculus kayanobori* Uchida, 1948: 322, fig. 8.

*Philopterus kayanobori* (Uchida, 1948); Hopkins and Clay 1952: 285; Price et al. 2003: 214.

*Philopteroides kayanobori* (Uchida, 1948); Mey 2004: 174.

**Type host.***Spizixos semitorques cinereicapillus* Swinhoe, 1871 – Collared finchbill bulbul (Pycnonotidae)

**Distribution.** Taiwan.

***Philopteroides sclerotifrons* (Tandan, 1955)**

*Philopterus sclerotifrons* Tandan, 1955: 417, figs 1–7. Price et al. 2003: 216.

*Philopteroides sclerotifrons* (Tandan, 1955); Mey 2004: 174.

**Type host.***Cinnyris asiaticus asiaticus* (Latham, 1790) – Purple sunbird (Nectariniidae)

**Distribution.** India.

***Philopteroides novaezelandiae* Mey, 2004**

*Philopteroides novaezelandiae* Mey, 2004: 174, figs 21–22c,d.

**Type host.***Acanthisitta chloris chloris* (Sparrman, 1787) – Rifleman (Acanthisittidae)

**Distribution.** South Island, New Zealand.

***Philopteroides xenicus* Mey, 2004**

*Philopteroides xenicus* Mey, 2004: 176, fig. 22a,b,e.

**Type host:***Xenicus longipes longipes* (Gmelin, 1789) – Bush wren (Acanthisittidae)

**Distribution.** South Island, New Zealand.

***Philopteroides cucphuongensis* Mey, 2004**

*Philopteroides cucphuongensis* Mey, 2004: 176, fig. 23, table 2.

**Type host:***Pycnonotus finlaysoni eous* Riley, 1940 – Stripe-throated bulbul (Pycnonotidae)

**Distribution.** Vietnam.

***Philopteroides flavala* Najer & Sychra, 2012**

*Philopteroides flavala* Najer & Sychra, 2012a: 39, figs 1, 2A–G, 5A–B.

**Type host:***Hemixos flavala* Blyth, 1845 – Ashy bulbul (Pycnonotidae)

**Distribution.** Vietnam.

***Philopteroides terpsiphoni* Najer & Sychra, 2012**

*Philopteroides terpsiphoni* Najer & Sychra, 2012b: 95, figs 6–12.

**Type host:** *Terpsiphone viridis* (Statius Müller, 1776) – African paradise-flyctacher (Monarchidae)

**Distribution.** Senegal.

### Philopteroides fuliginosus

Valim & Palma sp. n.

urn:lsid:zoobank.org:act:70A9F313-D518-4143-A683-E76F8168787B

http://species-id.net/wiki/Philopteroides_fuliginosus

Figs 5–6 , 11 , 21–24 , 34–35

#### Type host.

*Rhipidura fuliginosa placabilis* Bangs, 1921 – New Zealand fantail (Rhipiduridae)

#### Distribution.

New Zealand.

#### Description.

**Female.** Habitus as in Fig. 5 and head as in Fig. 11. Anterior setae 3 (*as3*) rigid, 0.04–0.05 long; anterior dorsal head setae (*ads*) peg-like, 0.02 long. Hyaline membrane with deep concavity and thick sclerotization of its margin, 0.03–0.04 long; anterior margin of the anterior dorsal plate deeply concave and ventral head plate more concave than the dorsal one (Fig. 34). Pterothorax with 8 posteromarginal setae on each side. Tergo-pleural plate II with a reduced postero-lateral projection overlapping segment III, tergo-pleural plate III without any projection. Tergocentral setae: II, 12–13 (plus 2 anterior setae); III, 15–16; IV, 13–18; V, 16–19; VI, 16–17; VII, 12–14; VIII, 6–12. Tergites VII–IX+X as in Fig. 21. Sternal setae (intraspecific variation in parentheses): II, SL-SL (plus 1–2 long setae laterad to sternal plate); III, LL-SLL (LSL-SLL); IV, LLSLL-LLSLL (LLSLL-LSLLL); V, LLSLL-LLSLL (LLSLL-LSLLL); VI, LLLL-LLLL (LLLLL-LLLL). Segments IV-VII with 1–4 long additional setae situated on soft tegument, between the sternal plate and the pleural setae. Paratergal setae (all long): II–III, 0; IV–V, 3; VI, 4 (rarely 3 on one side only); VII, 3–4; VIII, 1–2. Segments VI–VIII with an innermost dorsal seta on each tergite (included in the paratergal count), besides the postspiracular seta. Sternites III–VI with well-developed, large plates, roughly rectangular. Vulva with 7–9 medium long setae each side, and 4–6 small setae on the middle of the vulvar margin. Subgenital plate, inner genital sclerite, subvulval sclerites, and vulvar margin as in Fig. 22. Measurements (n = 8): HL 0.45–0.48, ANW 0.09–0.10, POL 0.20–0.22, POW 0.31–0.32, ADPL 0.18–0.20, ADPLL 0.15–0.16, ADPW 0.13–0.14, TRL 0.09–0.10, TRW 0.03–0.04, TW 0.43–0.46, PW 0.25–0.26, PTL 0.16–0.18, PTW 0.35–0.37, TPVL 0.21–0.23, AW 0.53–0.60, EWG 0.05–0.06; IWG 0.03–0.04, TL 1.37–1.49.

**Male.** Similar to female, except in dimensions and morphology of terminalia (Fig. 6). Pterothorax with 7–9 posteromarginal setae on each side. Tergocentral setae: II, 11–14 (plus 2 anterior setae); III, 10–18; IV, 10–17; V, 9–19; VI, 10–18; VII, 11–13; VIII, 7–8. Tergites VII–IX+X as in Fig. 23. Paratergal setae (all long): II–III, 0; IV–V, 2–4; VI–VII, 3–4; VIII, 2. Sternal plates well developed and entire on segments III–VI; subgenital plate with 4 proximal long setae and 2 distal postero-lateral long setae (Fig. 24). Genitalia: length of parameres 0.50 (Fig. 35). Measurements (n = 8): HL 0.42–0.46, ANW 0.09–0.10, POL 0.20–0.21, POW 0.28–0.30, ADPL 0.18–0.19, ADPLL 0.14–0.15, ADPW 0.11–0.12, TRL 0.09–0.10, TRW 0.03–0.05, TW 0.40–0.42, PW 0.24–0.25, PTL 0.14–0.16, PTW 0.32–0.35, TPVL 0.20–0.21, AW 0.47–0.53, GL 0.21–0.23, GW 0.06–0.07, TL 1.12–1.24.

#### Type specimens.

Holotype ♀ (MONZ AI. 030138), ex *Rhipidura fuliginosa placabilis*, NEW ZEALAND: Otaihanga, Paraparaumu, WN, 23.III.1996, N. Hyde col. Paratypes: 2♂ (MONZ AI.017297), 1♂, 1♀ (MZUSP #2886), same data as holotype; 2♂, 1♀ (MONZ AI.017295), same host species, NEW ZEALAND: Wallaceville Animal Research Centre, Upper Hutt, 4.IV.1974, D.M. Pearce col.; 1♂ (MONZ AI.017296), same host species, NEW ZEALAND: Little Barrier I., 1.IX.1977, C.R. Veitch col.; 1♂ (MONZ AI.017298), same host species, NEW ZEALAND: Days Bay, Wellington, 8.II.2003, E.W. Dawson col.

#### Additional specimens, non-types.

1♂ (MONZ AI.017290), ex *Rhipidura fuliginosa fuliginosa* (Sparrman, 1787), NEW ZEALAND: Jackson Bay, Westland, 6.VII.1969, W. Spiekman col.; 1♀ (MONZ AI.017291), NEW ZEALAND: Coutts I., Canterbury, 22.XII.1970, J.R. Jackson col.; 1♂, 1♀ (MONZ AI.017292), NEW ZEALAND: Nelson, 8.V.1972, B.A. Holloway col.; 2♂, 2♀ (MONZ AI.017293), NEW ZEALAND: Nelson, 22.V.1972, G. Kuschel col.; 1♀ (MONZ AI.017294), NEW ZEALAND: Franz Joseph Glacier, 1973–1974, P. Fletcher col.

#### Etymology.

The **s** pecies epithet is a noun in apposition derived from the species name of the host, and also refers to the dark colour of the lice.

#### Remarks.

Morphologically close to *Philopteroides macrocephalus* by having: anterior dorsal head setae (*ads*) peg-like; segment III without pleural setae; segment IV with 2 pleural setae; sternite VI without spine-like setae; setae of the male subgenital plate situated on the plate. However, both sexes of *Philopteroides fuliginosus* can be distinguished from those of *Philopteroides macrocephalus* by: (1) the hyaline margin with a shallower indentation (0.03–0.04mm) at its midline (compare Figs 11 and 12; 34 and 36); (2) the female tergal chaetotaxy and shape of tergites IX+X (Fig. 21); (3) the male subgenital plate (Fig. 24); and (4) the male genitalia (Fig. 34).

### Philopteroides macrocephalus

Valim & Palma sp. n.

urn:lsid:zoobank.org:act:968250A7-D7C5-470A-8BFE-5EBA96AC5314

http://species-id.net/wiki/Philopteroides_macrocephalus

Figs 7–8 , 12 , 25–28 , 36–37

#### Type host.

*Petroica macrocephala macrocephala* (Gmelin, 1789) – New Zealand tit or tomtit (Petroicidae)

#### Distribution.

New Zealand.

#### Description.

**Female.** Habitus as in Fig. 7, and head as in Fig. 12. Anterior setae 3 (*as3*) rigid, peg-shaped, 0.05–0.06 long; anterior dorsal head setae (*ads*) peg-like, 0.02 long. Hyaline membrane with deep concavity and thin sclerotization, 0.06–0.08 long; anterior dorsal plate slightly concave and ventral head plate deeply concave (Fig. 36). Pterothorax with 9 posteromarginal setae on each side. Tergo-pleural plate II with a much reduced postero-lateral projection overlapping segment III; tergo-pleural plate III without any projection. Tergocentral setae: II, 17–19 (plus 2 anterior setae); III, 23–25; IV, 25–27; V, 26–27; VI, 24–26; VII, 23–25; VIII, 12–14. Tergites VII–IX+X as in Fig. 25. Sternal setae: II, SL-LS; III, LLLLSL-LSLLLL; IV, LLLLSLL-LLSLLLL; V, LLLLLSLL-LLSLLLLL; VI, LLLLL-LLLLL (some specimens show slight variations from this symmetrical pattern). Segments II–VII with 3–5 additional long setae situated on the soft tegument, between the sternal plate and the pleural setae. Paratergal setae (all long): II–III, 0; IV, 5–6; V, 4–5; VI–VII, 4; VIII, 2. Tergites VI–VII with an innermost dorsal seta (included in the paratergal count), besides the postspiracular setae (not included in the count). Sternites III–VI with large, well-developed plates, roughly rectangular. Vulva with 9–11 medium to long setae each side, and 14–15 small setae in the middle of the vulvar margin. Subgenital plate, inner genital sclerite, subvulval sclerites, and vulvar margin as in Fig. 26, plus a variation of an additional female in Fig. 26a. Measurements (n = 7): HL 0.59–0.62, ANW 0.10–0.12, POL 0.28–0.30, POW 0.40–0.41, ADPL 0.24–0.25, ADPLL 0.20–0.21, ADPW 0.18–0.19, TRL 0.11–0.13, TRW 0.04–0.05, TW 0.58–0.64, PW 0.30–0.36, PTL 0.21–0.25, PTW 0.44–0.48, TPVL 0.25–0.27, AW 0.72–0.79, EWG 0.05–0.06, IWG 0.03–0.04, TL 1.85–2.13.

**Male.** Similar to female, except in dimensions and morphology of terminalia (Fig. 8). Pterothorax with 7–9 posteromarginal setae on each side. Tergocentral setae: II, 17–18 (plus 2 anterior setae); III, 17–19; IV, 22–24; V, 18–20; VI, 17–20; VII, 16–18; VIII, 10–12. Paratergal setae (all long): II–III, 0; IV–V, 4 (rarely 2 setae on one side only); VI–VII, 4–5 (rarely 3 setae on one side only); VIII, 2. Sternal plates III–VI well developed and entire; 4 long setae on the subgenital plate with only the anterior pair situated on the plate (Fig. 28). Genitalia: length of parameres 0.50 (Fig. 37). Measurements (n = 9): HL 0.55–0.57, ANW 0.10–0.12, POL 0.27–0.28, POW 0.37–0.39, ADPL 0.23–0.25, ADPLL 0.27–0.28, ADPW 0.16–0.17, TRL 0.10–0.12, TRW 0.04–0.05, TW 0.51–0.55, PW 0.28–0.30, PTL 0.17–0.20, PTW 0.42–0.44, TPVL 0.25–26, AW 0.62–0.65, GL 0.27–0.32, GW 0.07–0.08, TL 1.49–1.61.

#### Type specimens.

Holotype ♀ (MONZ AI.030122), ex *Petroica macrocephala macrocephala*, NEW ZEALAND: Haast Pass, 30.IX.1969, C.N. Challies col. (University of Canterbury). Paratypes: 3♂, 3♀ (MONZ AI.017278 & AI.017279), 1♂, 1♀ (MZUSP #2887), same data as holotype.

#### Additional specimens, non-types.

2♀ (MONZ AI.017281), ex *Petroica macrocephala dannefaerdi* (Rothschild, 1894), NEW ZEALAND: Penguin Creek, Snares Island, 29.III.1972, D.S. Horning & C.J. Horning cols.; 1♂ (MONZ AI.017282), NEW ZEALAND: Boat Harbour, Snares Is, 22.II.1975, H.A. Best col.; 1♂ (MONZ AI.017283), NEW ZEALAND: Station Point, Snares Island, 25.II.1977, D.S. Horning col.; 1♂ (MONZ AI.017284), NEW ZEALAND: Tern Point, Snares Islands, 10.I.1987, A. Tennyson col.; 1♂ (MONZ AI.017280), NEW ZEALAND: Snares Is, no date, CMu Av. 4635. 1♂ (MONZ AI.017277), ex *Petroica macrocephala*, NEW ZEALAND: Springs Junction, Maruia, 20.VIII.1966, J.R. Jackson col.

#### Etymology.

The **s** pecies epithet is a noun in apposition derived from the species name of the host, and also refers to the larger head of this louse species.

#### Remarks.

Morphologically close to *Philopteroides fuliginosus* by the features mentioned under that species above. However, both sexes of *Philopteroides macrocephalus* can be distinguished from those of *Philopteroides fuliginosus* by (1) the hyaline margin with a deeper incision (0.06–0.08mm) at its midline (compare Figs 11 and 12, Figs 34 and 36); (2) the female tergal chaetotaxy and shape of tergites IX+X (compare Figs 21 and 25); (3) the male subgenital plate (compare Figs 24 and 28); and (4) the male genitalia (compare Figs 35 and 37).

##### Key to the species of *Philopteroides* Mey, 2004 (adults only) *

*except *Philopteroides lineatus* (Giebel, 1874)

**Table:** 

1	Preantennal region short (POL ≤0.15–0.17) and broad (ANW ≥0.13–0.15); hyaline margin with a shallow concavity at midline (Figs 9–10, 30, 32)	*beckeri* species-group, 2
–	Preantennal region long (POL ≥0.20–0.28) and narrow (ANW ≤0.10–0.12); hyaline margin with a deep concavity at midline (Figs 11–12, 34, 36)	*mitsusui* species-group, 3
2	Sternite V without spine-like setae. Females: posterior setae on tergite IX+X situated outside plate (Fig. 13); subgenital plate with median setae (Fig. 14)	*Philopteroides beckeri* (Mey, 2004)
–	Sternite V with at least one pair of spine-like setae. Females: posterior setae on tergite IX+X situated on plate (Fig. 17); subgenital plate without median setae (Fig. 18)	*Philopteroides pilgrimi* Valim & Palma, sp. n.
3	Pleural setae present on segment III	*Philopteroides kayanobori* (Uchida, 1948)
–	Pleural setae absent on segment II	4
4	Pleural segment IV with only 1 setae on each side	*Philopteroides mitsusui* (Uchida, 1948)
–	Pleural segment IV with 2 or more setae on each side	5
5	Anterior dorsal setae (*ads*) peg-like (Figs 34, 36)	6
–	Anterior dorsal setae (*ads*) thin and rigid (Figs 30, 32)	10
6	Sternite VI with at least one pair of spine-like setae; without additional setae between pleural and sternal setae; sternites sexually dimorphic	*Philopteroides terpsiphoni* Najer & Sychra, 2012
–	Sternite VI without spine-like setae; with additional setae between pleural and sternal setae, situated on soft tegument; sternites not dimorphic	7
7	Males: anterior setae of the subgenital plate situated outside plate	8
–	Males: anterior setae of the subgenital plate situated on plate	9
8	Sternite V with at least one pair of spine-like setae. Male genitalia with a large trapezoidal medial sclerite on basal plate (as in fig. 22e in Mey 2004: 175)	*Philopteroides xenicus* Mey, 2004
–	Sternite V without spine-like setae. Male genitalia with a small roughly oval medial sclerite on basal plate (as in fig. 22d in Mey 2004: 175)	*Philopteroides novaezelandiae* Mey, 2004
9	Hyaline margin with a deep incision (0.06–0.08mm) at its midline (Figs 12, 36). Female tergite IX+X with an anterior and a posterior notch (Fig. 25). Lateral margin of the male subgenital plate with deep incisions (Fig. 28); male genitalia as in Fig. 37	*Philopteroides macrocephalus* Valim & Palma, sp. n.
–	Hyaline margin with a shallow incision (0.03–0.04mm) at its midline (Figs 11, 34). Female tergite IX+X without anterior notch and with an uneven posterior margin (Fig. 21). Lateral margin of the male subgenital plate with shallow incisions (Fig. 24); male genitalia as in Fig. 35	*Philopteroides fuliginosus* Valim & Palma, sp. n.
10	Females without central sternal plates, only with lateral rounded sternal plates on III–VI. Males without lateral rounded sternal plates	*Philopteroides sclerotifrons* (Tandan, 1955)
–	Females with central sternal plates, including lateral rounded sternal plates on III–VI (occasionally fused with the central plates). Males with lateral rounded sternites on at least segment III	11
11	With lateral rounded sternal plates on segment II. Females with a central sclerite plus two rounded lateral ones (separated from each other) on sternite VI	*Philopteroides flavala* Najer & Sychra, 2012
–	Without lateral sternal plates on segment II. Females with a unique central sclerite, and only vestiges of lateral rounded sternites (fused to the central sclerite) on sternite VI	*Philopteroides cucphuongensis* Mey, 2004

## Supplementary Material

XML Treatment for Philopteroides

XML Treatment for Philopteroides lineatus

XML Treatment for Philopteroides beckeri

XML Treatment for Philopteroides pilgrimi

XML Treatment for Philopteroides fuliginosus

XML Treatment for Philopteroides macrocephalus
